# Supplementary material for: Astrocytes Undergo Metabolic Reprogramming in the Multiple Sclerosis Animal Model
Source: Cells. 2023 Oct 19;12(20):2484. doi: 10.3390/cells12202484 (PMC10605171; doi:10.3390/cells12202484)
Supplement: Supplementary file 1 [file cells-12-02484-s001.zip › Supplementary file 2.pdf]

Supplementary figure 1

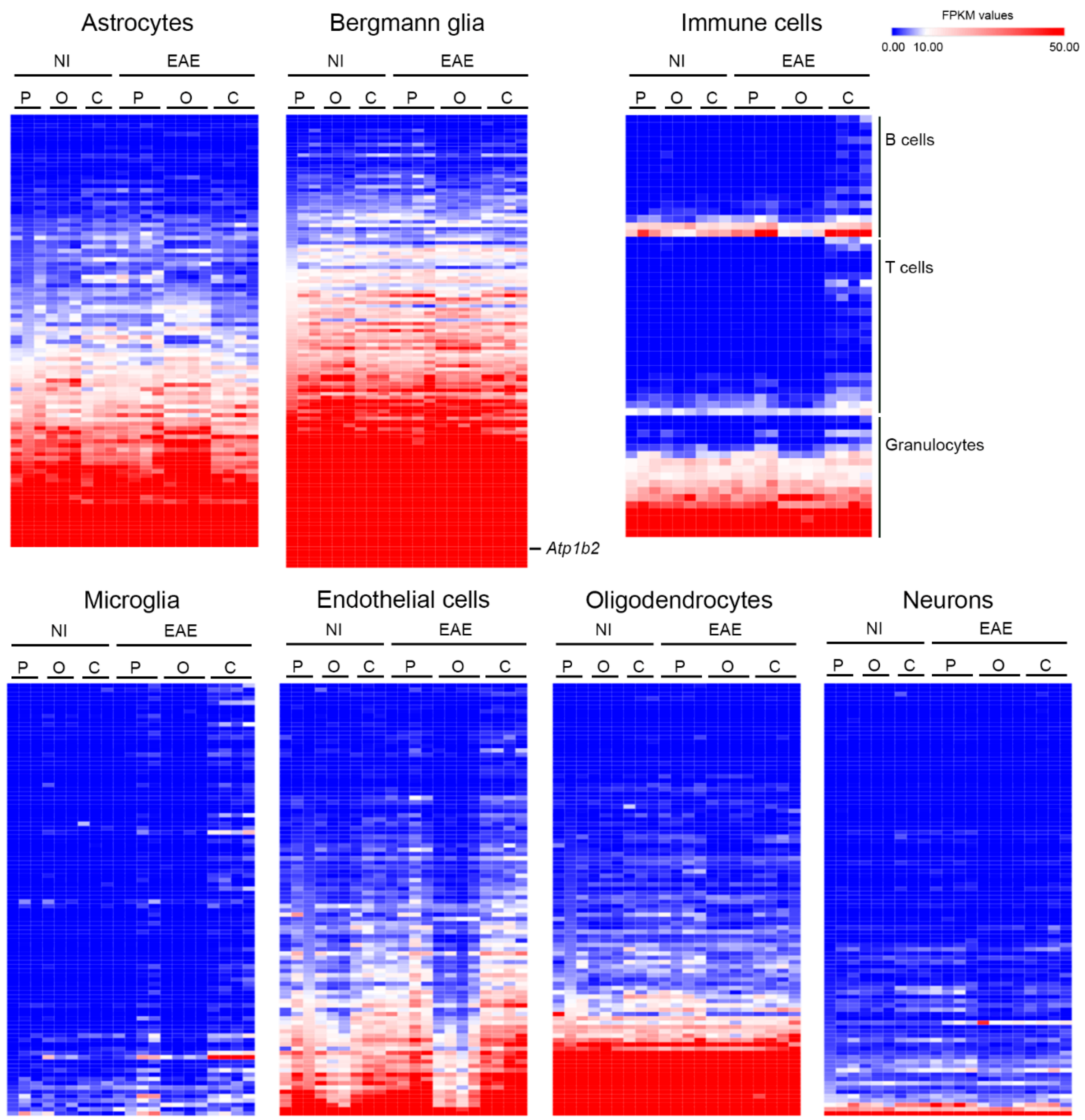

Figure S1. Expression levels of cell-type specific markers. Expression levels of genes associated with CNS cells (astrocytes, Bergmann glia, microglia, oligodendrocytes and neurons), with endothelial cells and different immune cell populations (B cells, T cells and granulocytes). The expression levels of *Atp1b2*, which was identified as the ACSA-2 epitope, used in the MACS technique to isolate the astrocytes, is evidenced.

Supplementary figure 2

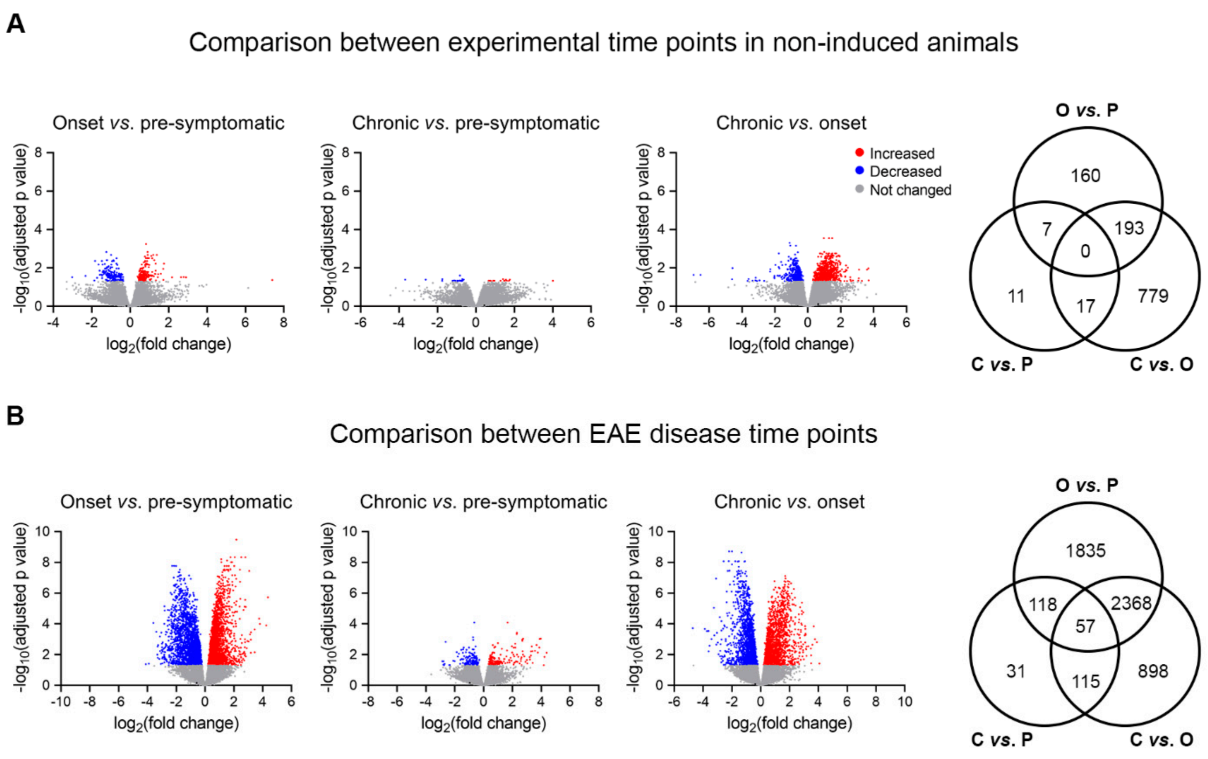

Figure S2. Volcano plots of differentially expressed genes. (A) Differentially expressed astrocytic genes for the comparisons between experimental time points in non-induced animals. (B) Differentially expressed genes for the comparisons between disease time points in EAE animals, before normalization to the respective non-induced group. Genes whose expression was not altered are represented in gray; significant underexpessed genes are represented in blue; significant overexpressed genes are represented in red. C – chronic time point; O – onset time point; P – pre-symptomatic time point.

Table S1. Primers' DNA sequences and annealing temperatures.

Gene	Primer sequence (5'→3')	Annealing temperature (ºC)
Amigo2	Fw: GAGGCGACCATAATGTCGTT<br>Rv: GCATCCAACAGTCCGATTCT	60
Fbln5	Fw: CTTCAGATGCAAGCAACAA<br>Rv: AGGCAGTGTCAGAGGCCTTA	58
Fkbp5	Fw: TATGCTTATGGCTCGGCTGG<br>Rv: CAGCCTTCCAGGTGGACTTT	60
Atp5b	Fw: GGCCAAGATGTCCTGCTGTT<br>Rv: GCTGGTAGCCTACAGCAGAAGG	60
Hspcb	Fw: GCTGGCTGAGGACAAGGAGA<br>Rv: CGTCGGTTAGTGGAATCTTCATG	60
Tbp	Fw: GGGAGAATCATGGACCAGAA<br>Rv: TTGCTGCTGCTGTCTTTGTT	55
Idh3g	Fw: GGCAATGCTCAAGCCAACTC<br>Rv: TGGAGGAATTGTTTGTTGTGAGG	60
Aldoc	Fw: GCCTGTTTGGTTAGGAGAGGA<br>Rv: CATGCTGCCTACGGACTCAT	60
Sdha	Fw: TGATGCTGTGGTTGTAGGCG<br>Rv: GATACCTCCCTGTGCTGCAA	60
Pfkm	Fw: GGTTTGGAAGCCTCTCCTCC<br>Rv: GGGTCATGATCCACTCTTGTAGT	60
